# CODA: Integrating multi-level context-oriented directed associations for analysis of drug effects

**DOI:** 10.1038/s41598-017-07448-6

**Published:** 2017-08-08

**Authors:** Hasun Yu, Jinmyung Jung, Seyeol Yoon, Mijin Kwon, Sunghwa Bae, Soorin Yim, Jaehyun Lee, Seunghyun Kim, Yeeok Kang, Doheon Lee

**Affiliations:** 1 Department of Bio and Brain Engineering, KAIST, 291 Daehak-ro, Yuseong-gu, Daejeon Republic of Korea; 2 Bio-Synergy Research Center, 291 Daehak-ro, Yuseong-gu, 305- 701 Daejeon Republic of Korea; 3 SD Genomics Co., Ltd., 619 Gaepo-ro, Gangnam-gu, Seoul Republic of Korea

**Keywords:** Cellular signalling networks, Data integration, Virtual screening

## Abstract

*In silico* network-based methods have shown promising results in the field of drug development. Yet, most of networks used in the previous research have not included context information even though biological associations actually do appear in the specific contexts. Here, we reconstruct an anatomical context-specific network by assigning contexts to biological associations using protein expression data and scientific literature. Furthermore, we employ the context-specific network for the analysis of drug effects with a proximity measure between drug targets and diseases. Distinct from previous context-specific networks, intercellular associations and phenomic level entities such as biological processes are included in our network to represent the human body. It is observed that performances in inferring drug-disease associations are increased by adding context information and phenomic level entities. In particular, hypertension, a disease related to multiple organs and associated with several phenomic level entities, is analyzed in detail to investigate how our network facilitates the inference of drug-disease associations. Our results indicate that the inclusion of context information, intercellular associations, and phenomic level entities can contribute towards a better prediction of drug-disease associations and provide detailed insight into understanding of how drugs affect diseases in the human body.

## Introduction

Drug development requires a great deal of money and time. It generally takes about 15 years and 1 billion US dollars and on top of that, more than 85% of drug candidates fail to be approved for the market^1^. High costs and attrition rates have to do with testing animals in preclinical trials and humans in clinical trials^2^. Recently, several *in silico* methods using biological networks, which represent biological systems with biological entities and their associations, have been developed for resolving these problems^3–5^. These *in silico* network-based approaches have attempted to facilitate the analysis of drug effects, in which constructing biological networks is deeply involved.

With the recent wealth of high-throughput data and literature being available, many databases collecting various types of biological associations such as protein-protein interactions, gene regulatory interactions, or gene-disease associations have been constructed^6–10^. Furthermore, the reconstruction of biological networks with the consideration of biological context has also been challenged by several studies^11–15^ because biological networks have heterogeneity depending on different biological contexts, especially anatomical contexts. For example, protein-protein interactions can be heterogeneous across tissues because of diverse gene and protein expressions in different anatomical contexts. Recently, many tissue-specific metabolic models or protein-protein interaction networks have been reconstructed by combining the network that has no anatomical context such as ReconX^9^ or BioGRID^8^ with expression data in the specific anatomical contexts^16^.

Although previous approaches have reconstructed anatomical context-specific networks successfully, there are still some imperfections in the previous networks in describing the human body system. Each context-specific network of previously constructed networks is isolated because intercellular associations are not included in the previous networks. Thus, these networks would not be appropriate for studying diseases that are related to the interplay of biological entities in different organs. In addition, although some previous research contained phenomic level entities such as biological processes or diseases in their networks, most of these works ignored the anatomical context information in their associations. However, biological associations of phenomic level entities are also dependent on anatomical contexts. For example, renin is associated with hypertension in kidney and sodium ion transport process is associated with hypertension in kidney.

In this study, we construct CODA (Context-Oriented Directed Associations) by integrating context-oriented directed associations. The CODA network covers both molecular level entities and phenomic level entities with anatomical contexts. In addition, our constructed network contains not only organ-specific intracellular associations but also intercellular associations which can be used in the analysis of diseases related to multi-organs. Using the CODA network, drug-drug target associations with anatomical contexts, and the network-based method using the proximity measures, we quantify the effects of drugs on diseases. From this analysis, we demonstrate the usefulness of unique characteristics of our network for the analysis of drug effects.

## Results

### Integration of context-oriented directed associations

**Figure 1:**
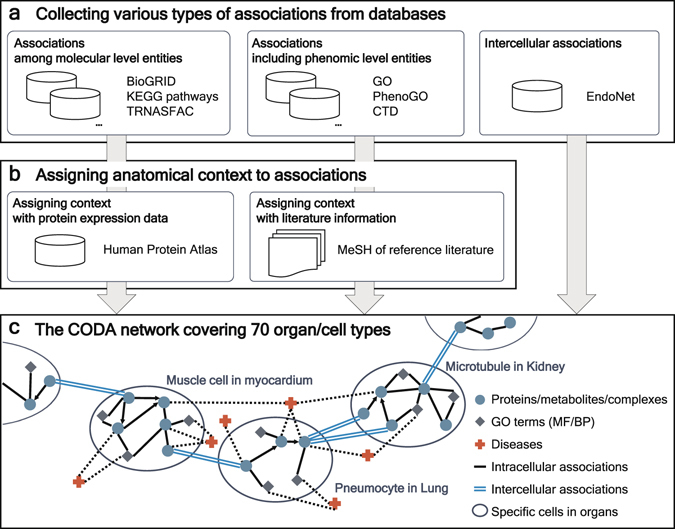
Overview of constructing the CODA network. (**a**) Associations including both molecular level entities and phenomic level entities are gathered from diverse databases, BioGRID, KEGG pathways, TRANSFAC, GO, PhenoGO, CTD, and EndoNet. All of the associations do not include anatomical contexts at first except for intercellular associations from EndoNet. (**b**) Anatomical contexts are assigned to associations among molecular level entities by using protein expression data from HPA. For associations including phenomic level entities, anatomical contexts are added to the associations using MeSH of their reference literature. Intercellular associations have anatomical context ab initio. (**c**) As a result, constructed CODA network consists of not only organ/cell type specific networks but also intercellular associations. Diverse associations among molecular level entities and phenomic level entities with anatomical context are contained in CODA network.

We build the CODA network by integrating various types of biological associations with their anatomical contexts. We start with collecting associations without anatomical contexts and then add anatomical contexts for these associations with expression data or scientific literature, with the exception of intercellular associations. Entire associations in CODA are stored in a modified version of Bio-Synergy Modeling Language (BSML) format^17^, which is presented in Methods and Supplementary Fig. 1, with selected ontologies and dictionaries (Supplementary Table 1). A method overview for constructing CODA network is illustrated in Fig. 1.

Various types of associations among molecular level entities without anatomical context are retrieved from three databases: signaling interactions from Kyoto Encyclopedia of Genes and Genomes (KEGG) pathways^7^, metabolic reactions from KEGG pathways, gene regulatory networks from KEGG pathways and TRANSFAC^10^, and protein-protein interactions from KEGG pathways and BioGRID^8^. Totally, 194,206 associations among 19,390 genes or proteins, 1,542 compounds, and 322 protein complexes are collected. We add anatomical context to these associations among molecular level entities by protein expression data from the Human Protein Atlas (HPA) data^16^. Similar to previous studies for reconstructing anatomical context-specific networks with expression data^11–13^, we assume that a protein-protein interaction would happen in parts of a human body if its two proteins or genes are expressed in the same parts of a human body. For directed associations such as activation or expression, our hypothesis is that the directed associations would take place in a body part if the left entity of the association is expressed in the body part in which there is a direction from the left entity to the right entity. In the case of metabolic reactions, we think that metabolic reactions would be present in a body part if enzymes of the metabolic reactions are expressed in the body part. To connect each anatomical context-specific network, we add intercellular associations from the EndoNet database^18^ which contains intercellular endocrine associations in the body. Detailed information about collected intercellular associations is described in the Methods section.

We also extract associations including phenomic level entities: associations between genes and Gene Ontology (GO) terms^19^, biological processes and molecular functions, from Gene Ontology Consortium; associations between genes and diseases from gene-disease associations of the Comparative Toxicogenomics Databases (CTD)^6^; associations between GO terms and diseases from PhenoGO^20^. The way we assemble these associations is described in the Methods section. We collect 195,496 gene-GO term associations, 18,524 gene-disease associations, and 5,966 GO term-disease associations without anatomical contexts. The reference literature of the associations is used to add anatomical contexts to these associations. Referring to the previous method^21^, we hypothesize that if the abstract of the reference of an association is related to an organ, the association would exist in the organ. Thus, an association is assigned to an organ if the name of the organ exists in Medical Subject Headings (MeSH)^22^ of the reference PubMed identification numbers (PMID) of the associations.

Finally, the CODA network contains a total of 5,864,692 associations with anatomical contexts among multifarious biological entities: 646,262 gene or protein entities, 83,546 compound entities, 7,447 protein complex entities, 9,800 biological processes, 4,206 molecular functions, and 3,586 diseases. Anatomical contexts in CODA network cover 70 organ-cell type pairs and blood. Detailed statistics of CODA are shown in Supplementary Data files: the number of associations with regard to anatomical contexts of their entities (Supplementary Data 1), the number of associations depending on their entity types (Supplementary Data 2), and the number of entities for each entity type (Supplementary Data 3).

### Inference of drug-disease relationships

We analyze the effects of drugs on diseases by calculating the extent of drug-disease associations. To this end, we exploit disease entities in the CODA network and drug-drug target associations from a chemical-gene association file of CTD (Supplementary Fig. 2). Similar to the above method that is used to allocate anatomical contexts to associations including phenomic level entities, the anatomical contexts revealing where the drug has effects on its target in the body are assigned to each drug-target association by using the MeSH of reference literature. We quantify the extent of the associations between a drug and a disease by calculating the score based on the average length of shortest paths between drug targets and the disease, which is analogous to the closest measure showing the best performance among various proximity measures as described in the previous work^4^ (see details in the Methods section). Based on the scores, we distinguish between unknown drug-disease associations and known drug-disease associations that are gathered from a chemical-disease association file of CTD by filtering associations having direct evidence.

### Using the CODA network performs better

**Figure 2:**
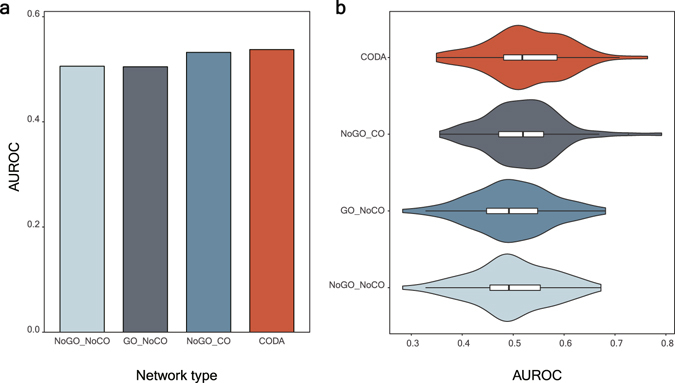
Performance comparison of CODA with other networks. (**a**) A bar graph for average AUROC values of inferring drug-disease relationships by using the four kinds of networks is shown. (**b**) A violin plot for AUROC values of inferring drug-disease relationships by using the four networks is revealed.

To demonstrate the utility of the CODA network, we compare the performances of inferring known drug-disease associations based on the average length of the shortest paths from targets of drugs to diseases in four networks: (i) a network without context information and excluding gene-GO, GO-disease associations (NoGO_NoCO in Fig. 2, the similar network used in the previous work)^4^, (ii) a network without context information and including gene-GO, GO-disease associations (GO_NoCO in Fig. 2), (iii) a network with context information and excluding gene-GO, GO-disease associations (NoGO_CO in Fig. 2), (iv) a network with context information and including gene-GO, GO-disease associations (CODA in Fig. 2), i.e., CODA. We calculate scores of 2,193 drugs for various types of 79 diseases after filtering (see Methods section and Supplementary Data 4). Figure 2 shows that using the CODA network exhibits the best average performance. Using the CODA network yields the best performance among the four networks in 35 diseases of whole the 79 diseases (Fig. 2 and Supplementary Data 5).

### Related organs for each disease category

To see whether the anatomical contexts of the CODA network are well allocated, we extract representative organs for nine disease categories used in our inference of drug-disease associations. Table 1 shows the top three organs that frequently appear in anatomical contexts of diseases in the categories. Table 1 reveals that many anatomical contexts for disease categories are assigned well: hippocampus, cerebellum, and cerebral cortex for nervous system diseases; myocardium and kidney for cardiovascular diseases; skeletal muscle and myocardium for musculoskeletal diseases; lung, lymph nodes, and bronchi for respiratory tract diseases; liver, kidney, and spleen for digestive diseases; skin for skin and connectivity tissue diseases; liver, kidney, and myocardium for nutritional and metabolic diseases. Entire anatomical contexts for whole diseases are listed in Supplementary Data 4.

**Table 1:** The representative organs of each disease category.

Disease category	Representative organs
Neoplasms	Liver, Lung, Colon
Nervous System Diseases	Hippocampus, Cerebellum, Cerebral Cortex
Hemic and Lymphatic Diseases	Bone Marrow, Spleen, Kidney
Cardiovascular Diseases	Myocardium, Kidney, Lung
Musculoskeletal Diseases	Adrenal Glands, ‘Muscle, skeletal’, Myocardium
Respiratory Tract Diseases	Lung, Lymph Nodes, Bronchi
Digestive System Diseases	Liver, Kidney, Spleen
Skin and Connective Tissue Diseases	Skin, Colon, Lung
Nutritional and Metabolic Diseases	Liver, Kidney, Myocardium

Representative organs mean the three most commonly assigned anatomical contexts for the diseases in the category.

### Inference of drug-disease relationships for each disease category

**Figure 3:**
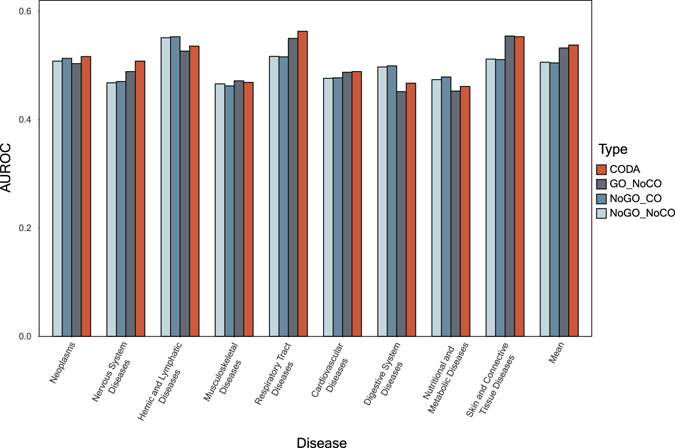
AUROC values for nine disease categories.

We identify the categories of diseases where the CODA network performs the best. To this end, we analyze AUROCs for the categories of diseases. Figure 3 shows the performance of predicting known drug-disease associations for nine disease categories of 79 diseases from MeSH tree. Using the CODA network yields the best performance in average and shows the best performance in four disease categories, neoplasms, cardiovascular diseases, nervous system diseases, and respiratory diseases. The categories where the CODA network shows the best predictions have relatively accurate representative organs (Table 1).

### Hypertension as a case study

**Figure 4:**
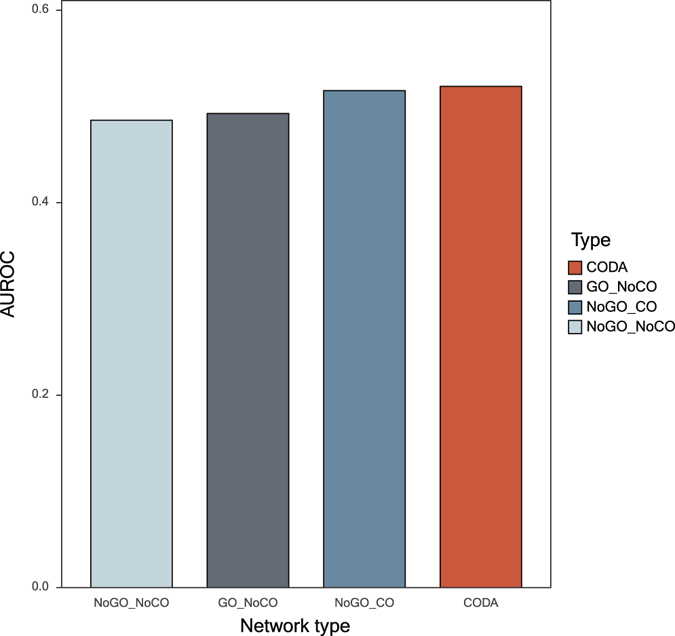
AUROC values of hypertension.

To investigate how the addition of anatomical context and phenomic level entities generates better predictions, we select hypertension as a case study. Hypertension is a medical condition in which the elevated blood pressure in arteries persists. Hypertension is chosen because of three reasons: (i) hypertension is known for being involved in multi-body parts such as kidney, myocardium, and adrenal glands, (ii) the mechanism of hypertension is associated with intercellular associations like renin-angiotensin system^23^, (iii) some GO terms like ‘sodium ion binding’ are related to the mechanism of hypertension^24^. These reasons are relevant to the novelties of our CODA network and thereby the above result of using the CODA network outperforms the results of using other networks in the case of hypertension as shown in Fig. 4. Detailed information about hypertension in the CODA network is presented in Supplementary Data 4 and the scores and ranks of drugs by the four networks for hypertension are exhibited in Supplementary Data 6.

### Examples of usefulness of anatomical context

**Figure 5:**
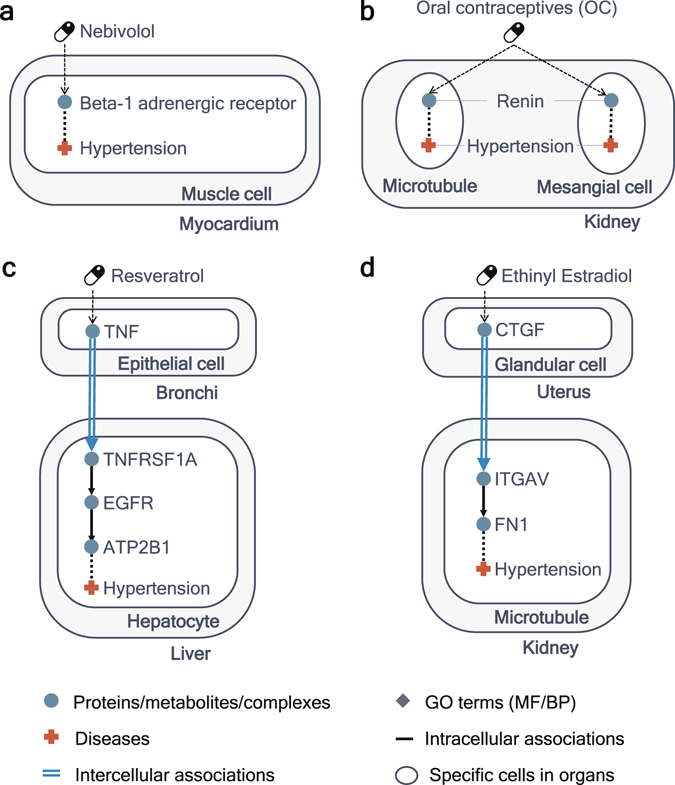
Usefulness of context information for inferring known drug-disease associations. (**a**) Illustration of the path from nebivolol to hypertension in the CODA network. Nebivolol affects beta-1 adrenergic receptor in muscle cell in myocardium and beta-1 adrenergic receptor is associated with hypertension in muscle cell in myocardium. (**b**) The shortest path from Oral contraceptives (OC) to hypertension in CODA. OC affects renin in kidney and renin is associated with hypertension in kidney. (**c**) One of the shortest paths from resveratrol’s targets to hypertension in CODA. Resveratrol has an effect on hypertension through intercellular associations. (**d**) One of the shortest paths from targets of ethinyl estradiol to hypertension. Ethinyl estradiol affects CTGF in glandular cell in uterus, CTGF in glandular cell in uterus affects ITGAV in microtubule in kidney, ITGAV in microtubule in kidney is associated with FN1 in microtubule in kidney, and FN1 in microtubule in kidney is associated with hypertension.

We find some instances of drugs which show the usefulness of anatomical context information in the identification of related drugs for hypertension. Nebivolol, which is clinically used for managing hypertension and has a known association with hypertension in a chemical-disease association file in CTD, acquires a high score only if we exploit context information. How nebivolol receives a high score in CODA network is described in Fig. 5a. Nebivolol is associated with beta-1 adrenergic receptor in muscle cells in myocardium, and beta-1 adrenergic receptor has an association with hypertension in muscle cells in myocardium. It is identical to known mechanisms of actions of nebivolol^25,26^. Oral contraceptives (OC) also has a known association with hypertension in CTD. There is a report that OC can cause significantly increased risks of hypertension^27,28^. OC gets a high score in CODA as it impacts on renin in both microtubules and mesangial cells of kidney and renin is connected to hypertension in kidney (Fig. 5b). It can be mechanisms of OC-induced hypertension though the exact mechanism is not verified yet.

Distinct from previous context-specific networks, the CODA network includes intercellular associations as well as intracellular associations. In our analysis, some drugs get higher scores in CODA on account of the shortest paths including intercellular associations. For instance, resveratrol, which is known to have an association with hypertension according to CTD, is connected to hypertension through the shortest paths including intercellular associations. One of them is as follows: resveratrol affects TNF in epithelial cells in bronchi, TNF in epithelial cells in bronchi affects TNFRSF1A in hepatocytes in liver, TNFRSF1A is associated with EGFR in hepatocytes in liver, EGFR is associated with ATP2B1 in hepatocytes in liver, and ATP2B1 in hepatocytes in liver is associated with hypertension (Fig. 5c). In the previous experiment, resveratrol treatment decreases the expression of inflammatory cytokines such as TNF whose activation is related to the development of pulmonary hypertension in MCT-treated rat^29,30^. It is closely akin to the shortest path from resveratrol to hypertension in the CODA network. Also, ethinyl estradiol gets a higher score by CODA because of the path including intercellular associations (Fig. 5d) and it is likewise known for affecting blood pressure^31^.

### Uses of phenomic level entities for inferring known drug-disease associations

**Figure 6:**
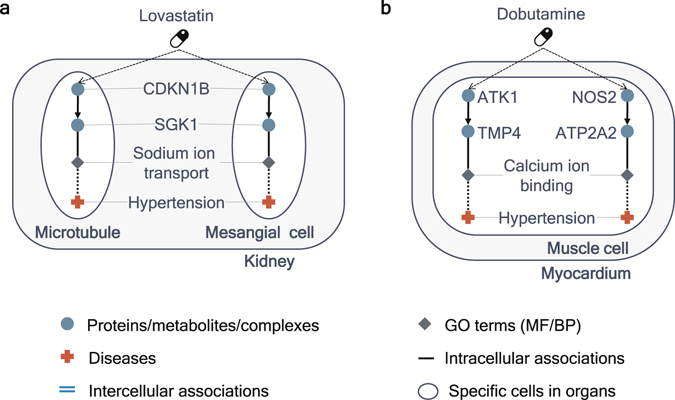
Uses of GO terms in the CODA network to infer known drug-disease associations. (**a**) The path from lovastatin to hypertension in CODA. Lovastatin is associated with hypertension through a biological process, sodium ion transport, in kidney. (**b**) The path from dobutamine to hypertension in CODA. Dobutamine is associated with hypertension through a molecular function, calcium ion binding, in myocardium.

Some drugs known for having associations with hypertension in a chemical-disease association file in CTD obtain higher scores by using entities of GO terms. For example, a score of lovastatin is higher in the CODA network than in a context-specific network without associations including GO terms. In the CODA network, lovastatin is connected to hypertension through these shortest paths: lovastatin affects CDKN1B in both microtubules and mesangial cells of kidney, CDKN1B is associated with SGK1 in microtubules and mesangial cells of kidney, SGK1 is associated with sodium ion transport in microtubules and mesangial cells of kidney, and finally sodium ion transport is associated with hypertension in microtubules and mesangial cells of kidney (Fig. 6a). Sodium ion transport, which is one of the biological processes in GO and is related to hypertension^24^, is known for mechanisms of actions of lovastatin for preventing hypertension^32^. Another example is dobutamine, which is associated with hypertension in CTD and gets higher scores using the networks with gene ontology entities than using the networks without gene ontology entities. The shortest paths from dobutamine to hypertension in the CODA network are like these: (i) dobutamine affects AKT1 in muscle cells in myocardium, AKT1 is associated with TMP4 in muscle cells in myocardium, TMP4 is associated with ‘calcium ion binding’ in muscle cells in myocardium, ‘calcium ion binding’ is associated with hypertension in muscle cells in myocardium; (ii) dobutamine affects NOS2 in muscle cells in myocardium, NOS2 is associated with ATP2A2 in muscle cells in myocardium, ATP2A2 is associated with ‘calcium ion binding’ in muscle cells in myocardium, ‘calcium ion binding’ is associated with hypertension in muscle cells in myocardium (Fig. 6b). Dobutamine may be related to the increase of calcium^33^, which is detected in hypertension patients^24^. Thus, these paths can be a possible mechanism of that how dobutamine affects hypertension.

### Novel drug-disease associations by CODA

**Figure 7:**
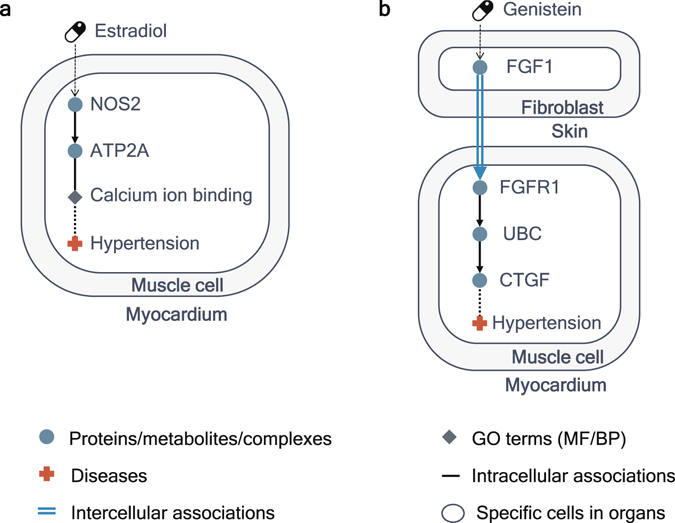
Inference of novel drug-disease associations by using CODA. (**a**) The path from estradiol to hypertension in muscle cell in myocardium in CODA. This path includes ‘calcium ion binding’, a molecular function, which is one of the novelties of CODA network. (**b**) The path from genistein to hypertension. Genistein affects FGF1 in fibroblast in skin at the first time and finally has effects on hypertension in muscle cell in myocardium through an intercellular association from FGF1 in fibroblast in skin to FGFR1 in muscle cell in myocardium.

Several drugs, which receive relatively high scores in CODA but are not associated with hypertension in CTD, can be new drug-disease associations like novel candidates of drug repurposing or side effects. Estradiol, 17-beta-isomer of estradiol, has the shortest path from its target NOS2 to hypertension through NOS2, ATP2A2, and ‘calcium ion binding’ in muscle cells in myocardium (Fig. 7a). Although estradiol is not contained in the list of drugs related to hypertension from CTD, there is previous research showing that estradiol can reduce the blood pressure^34^. Genistein obtains high scores with context information because it is connected to hypertension across body parts through its target in CODA: genistein affects FGF1 in fibroblasts in skin, FGF1 in fibroblasts in skin affects FGFR1 in muscle cells in myocardium, FGFR1 is associated with UBC in muscle cells in myocardium, UBC is associated with CTGF in muscle cells in myocardium, and finally CTGF is associated with hypertension in muscle cells in myocardium (Fig. 7b). Although the mechanism of action of genistein has not been identified yet, genistein is known for having the potentiality for being used in treating hypertension^35^ and this path can be the mechanisms of actions of genistein.

## Discussion

Here, we construct the CODA network and employ the context-specific network for the analysis of drug effects. Every entity in our network has an anatomical context. The above results manifest increased performances in the prediction of drug-disease associations with anatomical context information and imply that anatomical contexts can be used to predict effects of drugs in the body. The network used in our analysis is distinguished from other context-specific networks in two ways: (i) the inclusion of intercellular associations, (ii) the presence of phenomic level entities. Past context-specific networks did not include intercellular associations and, consequently, they are not appropriate for studying multi-organ diseases. Our analysis reveals that the existence of intercellular associations is indeed useful to explore the association between drugs and hypertension whose pathophysiology is related to several body parts and intercellular interactions among them. In addition, most of the previous context-specific networks only contain molecular level entities such as genes or metabolites and therefore, these networks cannot be directly used to predict the effects of drugs on diseases. Furthermore, our results indicate that the inclusion of GO terms, biological processes and molecular functions, has the potential for increasing performance in the inference of drug-disease associations.

Our analysis suggests that our network can be exploited to understand mechanisms of actions of drugs in the human body. As presented in the above results, the CODA network enables us to better understand how drugs affect diseases by searching the paths from the drugs to the diseases on the network with the consideration of anatomical contexts: for example, a drug affects its target in a liver, the target in liver impacts on a gene in kidney, the gene in kidney influences a biological process in kidney, and finally the biological process in kidney has an effect on a disease in kidney. This kind of analysis can give researchers a better interpretation of drug effects in the body.

In several areas, our network can become more advanced and accurate. Assigning contexts more precisely can produce better results. For example, the assignment of anatomical contexts for gene-disease associations, gene-GO term associations, GO term-disease associations, and drug-target associations from literature was performed in the abstract level and it could bring out false positive associations. If the allotment of an anatomical context is fulfilled in the sentence level, some of false positives could be filtered out. Addition of other expression data can not only reduce false positives but also extend the coverage of body parts in CODA. Other entity types like symptoms and other association types such as disease-disease associations or GO-symptom associations also can be added and it can give us better understanding of drug effects.

The analysis of drug effects in this study can be improved in some portions as well. Though we preserve original association types such as activation or inhibition in the CODA network, types of associations that can be used to decide whether a drug brings about a disease as a side effect or treats a disease^5^ are not considered in this study. Also, we do not discriminate associations among molecular level entities and associations including phenomic level entities even though these associations are in different scale. In further studies, we plan to analyze drug effects in our network with different edge weights depending on edge types.

## Methods

### BSML format

Every biological association from various public resources is transformed to Bio-Synergy Modeling Language (BSML) format as described in the Supplementary Fig. 1, which was initially introduced in the previous work^17^. This BSML format was invented to represent biological interactions with a rule-based modeling, which basically consists of a triplet (‘object’, ‘association’, ‘object’). Each ‘object’ is comprised of three elements, i.e. ‘function’, ‘entity’ and ‘anatomy’. In this study, we assign ‘abundance’ to a ‘function’ term for every ‘object’ because all of the ‘objects’ that we collect from the source databases do not contain any kind of ‘function’. In this work, an ‘entity’ is a molecular level entity or a phenomic level entity, i.e. molecular level entities such as genes, metabolites, or complexes and phenomic level entities such as GO terms or diseases. A related anatomical context of ‘entity’ is assigned as an ‘anatomy’ term, such as cell types and organs. In this study, rather than employing the ‘namespace’ term, which was introduced in the previous BSML format^17^, whole ‘entity’, ‘anatomy’, and ‘organismal context’ are mapped to a corresponding ontologies and dictionaries, which are collected and constructed by us: genes and proteins to Entrez gene ID^36^; metabolites in metabolic reactions and compounds to the STITCH (Search Tool for Interacting Chemicals)^37^; phenomic level entities such as GO terms and diseases are mapped to Unified Medical Language System (UMLS) ID^38^; organisms in the organismal context and organs and cells in the anatomical context to Medical Subject Headings (MeSH)^22^. The ontologies and dictionaries we used are described in Supplementary Table 1.

### Associations among molecular level entities without anatomical context

We collect intracellular association data among molecular level entities covering diverse types from KEGG pathways^7^, TRANSFAC^10^, and BioGRID^8^. KEGG pathways were downloaded using KEGG Markup Language (KGML) files for 286 human pathways by using KEGGgraph R package^39^. We include eight types of relations and metabolic reactions whose entries are genes, metabolites, or complexes. Among 16 relation types in KEGG pathways, we exclude some relation types by some criteria: the number of relations are small, the meaning or direction of relations are ambiguous, the relation types are overlapped with ‘reaction’ in KEGG, or no mapped relations to our association dictionary. As a result, eight relation types, ‘methylation’, ‘ubiquitination’, ‘N/A’. ‘state change’, ‘missing interaction’, ‘indirect effect’, ‘compound’, and ‘hidden compound’, are filtered out.

We also extract gene regulatory associations from TRANSFAC resulting in 8,059 regulations between 837 regulators and 2,657 target genes. The regulators include 534 transcription factors and 303 microRNAs, and each type of regulators is involved in 5,611 and 2,448 regulations for 1,983 and 1,128 target genes respectively.

In order to retrieve a large-scale human protein-protein interaction network, protein-protein interactions are extracted from BioGRID. To consider only physical interactions, we use interactions which have interaction type codes: psi-mi:“MI:0407” (direct interaction), psi-mi:“MI:0915” (physical association), psi-mi:“MI:0914” (association), psi-mi: “MI:0403” (colocalization). All interactions are represented as “interact” in the association ontology. BioGRID uses Entrez identifier to refer to all proteins that can be directly mapped to the BSML gene/protein ontology. The total number of unique PPIs from BioGRID is 157,248.

### Adding anatomical context to associations among molecular level entities

To construct anatomical context-specific molecular interactions, we combine collected intracellular interactions and protein expression data from the Human Protein Atlas (HPA)^16^. We download protein expression profiles for normal tissue data. Proteins in HPA having Ensembl gene identifier are mapped to our ontology which is based on Entrez gene ID using “org.Hs.eg.db” package in R. Tissue and cell types, which are not mapped to our ontology, are filtered out. Finally, we use expression data from 70 organ-cell type pairs (Supplementary Data 1) and construct anatomical context-specific network among molecular level entities for these 70 organ-cell type pairs with the addition of the blood-specific network for the inclusion of intercellular associations as described in the next section.

### Intercellular associations

Intercellular molecular associations are extracted from EndoNet^18^. EndoNet is a manually curated database for intercellular regulatory interactions, which are mediated by secreted messengers. The secreted messengers are mostly hormones, but they also include growth factors, cytokines, and so on. EndoNet contains a secreted messenger, a receptor, and a cell/tissue/organ as entity types. It provides interactions from secreted messengers to receptors and the most of these interactions are intercellular interactions. The locations where the interactions occur are also provided. Locations consist of cells and organs and we take account of the locations as anatomical contexts. The intercellular associations in EndoNet occur directly or via blood. Since the 70 organ-cell type pairs do not include blood, we add the blood-specific network, which contains whole associations among molecular level entities, to CODA network as a channel of intercellular associations. In the case of the associations occurring directly between one location and another location, the interaction is translated to an activation from the location to the other location like ‘a hormone < one location > activate a receptor < another location >’. In the case of the associations occurring via blood, to precisely describe the bindings, an interaction is divided into two processes: a translocation from one location to blood, and an activation from blood to another location. For example, ‘a messenger < one location > translocate a messenger < blood >’, and ‘a messenger < blood > activate a receptor < another location >’. Messengers, receptors, and locations are mapped to our ontology with exact matching and manual curation and the intercellular associations that occur in the 70 organ-cell type pairs from HPA and blood are included in our CODA network. Total 5,925 intercellular associations are extracted from EndoNet.

### Associations including phenomic level entities without anatomical context

Gene–disease associations are retrieved from the Comparative Toxicogenomics Database (CTD)^6^. We filtered gene-disease associations which have direct evidence (marker/mechanism or therapeutic). CTD uses Entrez identifiers to refer to genes/proteins and MeSH identifiers to refer to diseases. We map genes and diseases with our constructed ontologies. In order to connect genes to GO terms, we collect genes and their functional annotation from the Gene Ontology database^19^. We extract biological processes and molecular functions among functional annotation categories. Genes are represented as Entrez Gene IDs and GO terms are mapped to UMLS IDs. To collect GO term-disease associations, we use PhenoGO^20^. PhenoGO is a database providing phenotypic contexts (the disease, cell type, tissue, and organ) to gene ontology terms. We only use associations which have disease context to biological processes and molecular functions. PhenoGO uses UMLS or MeSH identifier to refer to diseases. Thus, we convert MeSH IDs to UMLS IDs and map to BSML disease ontology. GO terms are mapped to our ontology using UMLS IDs.

### Assigning anatomical context to associations including phenomic level entities

To assign anatomical contexts to associations including phenomic level entities, we refer to the previous work^21^. Similar to the method in the previous study, we use reference PubMed IDs, which correspond to the papers from which the associations are generated, of each association from CTD, GO, and PhenoGO to add anatomical contexts to the associations. PubMed is manually indexed with Medical Subject Headings (MeSH) vocabularies from National Library of Medicine. The MeSH terms for organs from the reference literature of associations are assumed to determine the context information of the associations. To use these associations with anatomical contexts in our analysis, we make two adjustments. Firstly, organs that are not contained in the 42 organs or blood are discarded. Secondly, since it is hard to get the anatomical context at the cell type level in this way, we duplicate associations in organs for each cell type in the 70 organ-cell type pairs.

### AUROC analysis

To quantify the degree of drug-disease associations in the network, we refer to the proximity measure in the previous study^4^. We calculate a score between a drug *R* having drug targets *r*_*i*_ and a disease *D* based on the inverse value of the average length of the shortest paths from its target to the disease in four network we used through equation (1).

1 $$Score(R,D)=\frac{1}{\frac{1}{{N}_{r}}{\sum }_{i=1}^{{N}_{r}}d({r}_{i},D)}$$

where *N*_*r*_ is the number of targets of the drug *R*. We identify the shortest path based on Breadth-first search algorithm in R project. To avoid an infinite loop, the maximum length of the shortest path is restricted to 30. In this analysis, the protein-protein interactions are regarded as bi-directional and other association types are considered as having a direction from a left entity to a right entity. Metabolic reactions are excluded in this analysis because metabolites are not connected to any GO term and disease in our network.

We calculate scores between drugs and diseases in four networks: (i) a network without anatomical context information and removing associations including GO term-related associations, (ii) a network without anatomical context information and including GO term-related associations, (iii) a network with anatomical context information and removing GO term-related associations, (iv) a network with anatomical context information and including GO term-related associations. Only the associations having direct evidence in a chemical-gene association file from CTD were used to calculate scores between drugs and diseases. To obtain the scores in the network with anatomical context information, we add anatomical contexts to drug-target associations with the same method, which is applied to assign anatomical contexts to the associations including phenomic level entities, by using MeSH of abstracts where the associations come from.

Based on the calculated scores between drugs and diseases, we use Area Under Receiver Operating Characteristic (AUROC) values to validate whether our CODA network can increase the performance of inferring known drug-disease associations. We identify drugs affecting diseases for the diseases which have more than ten associations with drugs in a chemical-disease association file. We did not predict drugs for otorhinolaryngologic diseases (C09 in MeSH trees), eye diseases (C11 in MeSH trees), male urogenital diseases (C12 in MeSH trees), female urogenital diseases and pregnancy complications (C13 in MeSH trees), ‘congenital, hereditary, and neonatal diseases and abnormalities diseases’ (C16 in MeSH trees), animal diseases (C22 in MeSH trees), ‘pathological conditions, signs and symptoms’ (C23 in MeSH trees), and chemically-induced disorders (C25 in MeSH trees) because of some reasons: the current version of our network does not include the eye, nose, and throat, cannot discriminate genders, and does not delineate animal diseases; ‘pathological conditions, signs and symptoms’ are at a quite different level compared to other disease categories; our network illustrates a status in which no drugs or chemicals are taken and some diseases in the chemically-induce disorders such as ‘drug-related side effects and adverse reactions’ do not have any explicit drug and disease information so that we cannot determine specific disease based on the disease name. AUROC values are calculated with receiver operating characteristic curves which are drawn by the true positive rate and false positive rate with different thresholds.

## Electronic supplementary material

Supplementary Information

Dataset 1

Dataset 2

Dataset 3

Dataset 4

Dataset 5

Dataset 6
