# Author Correction: CODA: Integrating multi-level context-oriented directed associations for analysis of drug effects

**DOI:** 10.1038/s41598-018-19511-x

**Published:** 2018-01-17

**Authors:** Hasun Yu, Jinmyung Jung, Seyeol Yoon, Mijin Kwon, Sunghwa Bae, Soorin Yim, Jaehyun Lee, Seunghyun Kim, Yeeok Kang, Doheon Lee

**Affiliations:** 1 Department of Bio and Brain Engineering, KAIST, 291 Daehak-ro, Yuseong-gu, Daejeon Republic of Korea; 2 Bio-Synergy Research Center, 291 Daehak-ro, Yuseong-gu, 305- 701 Daejeon Republic of Korea; 3 SD Genomics Co., Ltd., 619 Gaepo-ro, Gangnam-gu, Seoul Republic of Korea

Correction to: *Scientific Reports* 10.1038/s41598-017-07448-6, published online 08 August 2017

The original version of this Article contained a typographical error in the spelling of the author Doheon Lee, which was incorrectly given as Dohoen Lee. This has now been corrected in the PDF and HTML versions of the Article, and in the accompanying Supplementary Information file.

