# Publisher Correction: CODA: Integrating multi-level context-oriented directed associations for analysis of drug effects

**DOI:** 10.1038/s41598-018-22351-4

**Published:** 2018-03-06

**Authors:** Hasun Yu, Jinmyung Jung, Seyeol Yoon, Mijin Kwon, Sunghwa Bae, Soorin Yim, Jaehyun Lee, Seunghyun Kim, Yeeok Kang, Doheon Lee

**Affiliations:** 1 Department of Bio and Brain Engineering, KAIST, 291 Daehak-ro, Yuseong-gu, Daejeon Republic of Korea; 2 Bio-Synergy Research Center, 291 Daehak-ro, Yuseong-gu, 305- 701 Daejeon Republic of Korea; 3 SD Genomics Co., Ltd., 619 Gaepo-ro, Gangnam-gu, Seoul Republic of Korea

Correction to: *Scientific Reports* 10.1038/s41598-017-07448-6, published online 08 August 2017

An error occurred after publication resulting in the inadvertent omission of the Article and Volume numbers from the PDF version of this Article. This error has now been corrected in the PDF version of the Article; the HTML version was correct from the time of publication.

