# Supplementary material for: CODA: Integrating multi-level context-oriented directed associations for analysis of drug effects
Source: Sci Rep. 2017 Aug 8;7:7519. doi: 10.1038/s41598-017-07448-6 (PMC5548804; doi:10.1038/s41598-017-07448-6)
Supplement: Supplementary file 1 — Supplementary Information [file 41598_2017_7448_MOESM1_ESM.doc]

**Supplementary Information for**

**CODA: Integrating multi-level context-oriented directed associations for analysis of drug effects**

Hasun Yu^1,2,§^, Jinmyung Jung^1,2,§^, Seyeol Yoon^1,2^, Mijin Kwon^1,2^, Sunghwa Bae^1,2^, Soorin Yim^1,2^, Jaehyun Lee^1,2^, Seunghyun Kim^1,2^, Yeeok Kang^3^, Doheon Lee^1,2,*^

^1^ Department of Bio and Brain Engineering, KAIST, 291 Daehak-ro, Yuseong-gu, Daejeon, 305- 701, Republic of Korea

^2^ Bio-Synergy Research Center, 291 Daehak-ro, Yuseong-gu, Daejeon, 305- 701, Daejeon, Republic of Korea

^3^ SD Genomics Co., Ltd., 619 Gaepo-ro, Gangnam-gu, Seoul, Republic of Korea

*Corresponding author

^§^Co-first authors

**Inventory**

Supplementary Tables

Supplementary Figures

Supplementary References

**Supplementary Table**s

| **Type** | **Detailed type** | **Reference DB** |
| --- | --- | --- |
| Entity | Gene (Protein) | Entrez ID |
|  | Metabolite and compound | STITCH |
|  | Biological process | UMLS |
|  | Molecular function |  |
|  | Disease |  |
| Association | Association | Text from LMU |
| Anatomical context | Cell | MeSH |
|  | Organ |  |
| Organismal context | Organism |  |

Supplementary Table 1: Ontologies and dictionaries used in BSML format. In our CODA network, all of associations are stored in BSML format^1^. All of entities, associations, and contexts are mapped with selected ontologies and dictionaries: genes are mapped to Entrez IDs^2^, metabolites and compounds are mapped to STITCH^3^, GO terms and diseases are mapped to UMLS IDs^4^, associations are mapped to previously described association dictionaries^5^, and contexts are mapped to MeSH IDs^6^.

**Supplementary Figures**


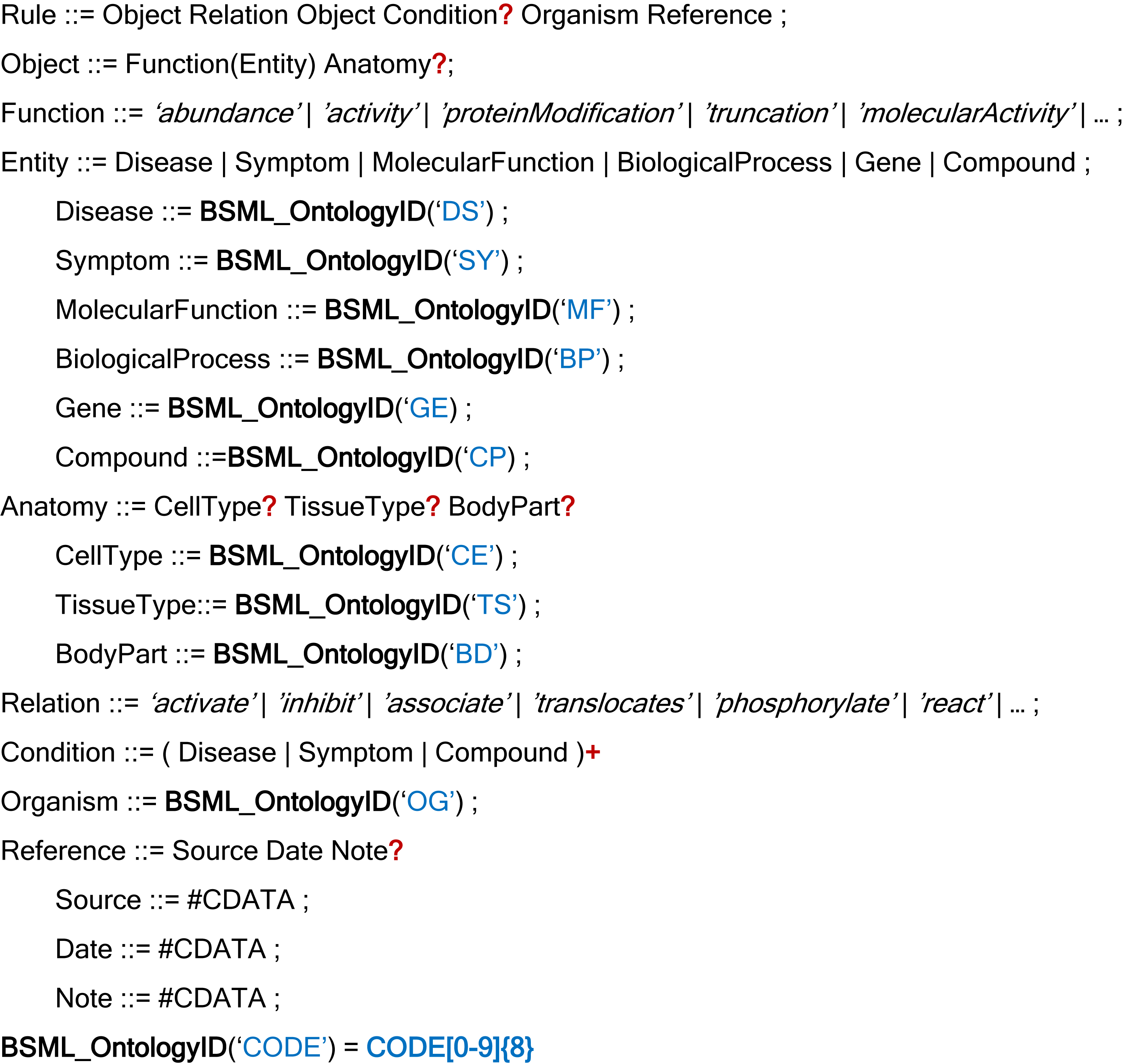


**Supplementary Figure 1**: **Bio synergy modeling language.** Every biological interaction from various public resources is transformed to BSML format, which has been updated from the firstly introduced BSML format in Hwang et al work**.** This BSML format was invented to represent biological interactions with rule-based modeling, which basically consists of triplet (‘object’, ‘association’, ‘object’). Each ‘object’ is comprised of three elements, i.e. ‘function’, ‘entity’ and ‘anatomy’. An ‘entity’ can be one of three level entities, i.e. molecule level (gene and compound), GO term level (molecular function and biological process) and phenotype level (disease and symptom) entities. Every ‘entity’ is mapped to an instance in the corresponding BSML ontology. A ‘function’ term indicates status of an entity, such as ‘abundance’, ‘activity’ and so on. A related anatomical context of an entity is assigned to ‘anatomy’ terms, such as cell, tissue and organ. An ‘association’ term demonstrates a relationship between a left object and a right object. If a rule occurs under a specific condition, it is represented as a ‘condition’ term, such as disease, symptom and compound. We also depicts the reference of a rule with information of the source, date and additional note. In this study, we assigned ‘abundance’ to ‘function’ term for every ‘object’ because the ‘objects’ we treated did not need other kinds of functions.


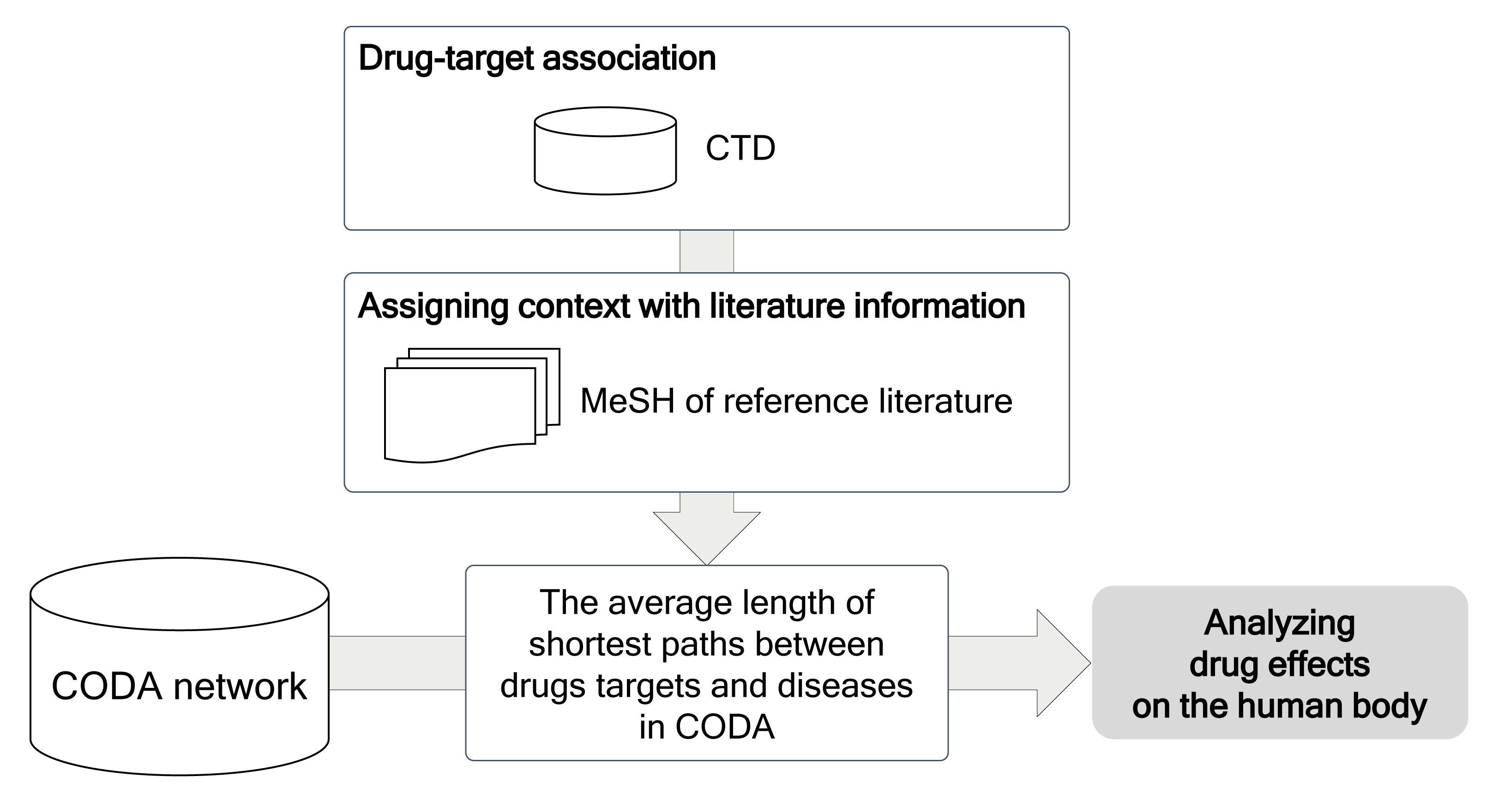


Supplementary Figure 2: A framework for analyzing effects of drugs using the CODA network. To this end, we also assign anatomical contexts to drug-target associations. Drug-target associations are extracted from CTD and anatomical contexts are added to them based on MeSH of the abstracts of the reference. For a drug and a disease, we calculate the average length of shortest paths between targets of the drug and the disease in the CODA network. Based on this value, we measure the effects of drugs on diseases.

**Supplementary References**

1 Hwang, W., Choi, J., Jung, J. & Lee, D. in *Proceedings of the 7th international workshop on Data and text mining in biomedical informatics* 19-20 (ACM, San Francisco, California, USA, 2013).

2 Maglott, D., Ostell, J., Pruitt, K. D. & Tatusova, T. Entrez Gene: gene-centered information at NCBI. *Nucleic Acids Res* **33** (2005).

3 Szklarczyk, D. *et al.* STITCH 5: augmenting protein-chemical interaction networks with tissue and affinity data. *Nucleic Acids Res* **44**, D380-384, doi:10.1093/nar/gkv1277 (2016).

4 Bodenreider, O. The Unified Medical Language System (UMLS): integrating biomedical terminology. *Nucleic Acids Res* **32**, D267-270, doi:10.1093/nar/gkh061 (2004).

5 Fundel, K., Kuffner, R. & Zimmer, R. RelEx--relation extraction using dependency parse trees. *Bioinformatics* **23**, 365-371, doi:10.1093/bioinformatics/btl616 (2007).

6 Coletti, M. H. & Bleich, H. L. Medical subject headings used to search the biomedical literature. *J Am Med Inform Assoc* **8**, 317-323 (2001).
